# Interorgan Metabolism of Ganglioside Is Altered in Type 2 Diabetes

**DOI:** 10.3390/biomedicines10123141

**Published:** 2022-12-06

**Authors:** Irma Magaly Rivas Serna, Michelle Beveridge, Michaelann Wilke, Edmond A. Ryan, Michael Thomas Clandinin, Vera Christine Mazurak

**Affiliations:** 1 Division of Human Nutrition, Department of Agricultural, Food and Nutritional Science, Li Ka Shing Centre for Health Research Innovation, University of Alberta, Edmonton, AB T6G 2P5, Canada; 2 Department of Medicine, University of Alberta, Edmonton, AB T6G 2R7, Canada

**Keywords:** GD1, GM3, GD3, postprandial period, diabetes, LC/triple quad MS, chylomicrons, plasma

## Abstract

GM3 is implicated in cell signaling, inflammation and insulin resistance. The intestinal mucosa metabolizes ganglioside and provides gangliosides for uptake by peripheral tissues. Gangliosides downregulate acute and chronic inflammatory signals. It is likely that transport of intestinal derived gangliosides to other tissues impact the same signals characteristic of inflammatory change in other chronic conditions such as Type 2 Diabetes (T2DM). The postprandial ceramide composition of GM3 and other gangliosides in plasma and chylomicrons has not been examined in T2DM. The present study assessed if diet or T2DM alters ganglioside components in plasma and chylomicrons secreted from the intestinal mucosa after a meal. GD1, GD3, and GM3 content of chylomicrons and plasma was determined by LC/triple quad MS in non-diabetic (control) and T2DM individuals in the fasting and postprandial state after 2 days of consuming a low or high fat diet in a randomized blinded crossover design. Diet fat level did not alter baseline plasma or chylomicron ganglioside levels. Four hours after the test meal, plasma monounsaturated GD3 was 75% higher, plasma saturated GD3 was 140% higher and plasma polyunsaturated GM3 30% lower in diabetic subjects compared to control subjects. At 4 h, chylomicron GD1 was 50% lower in T2DM compared to controls. The proportion of d34:1 in GD3 was more abundant and d36:1 in GD1 less abundant in T2DM compared to control subjects at 4 h. The present study indicates that T2DM alters ceramide composition of ganglioside available for uptake by peripheral tissues.

## 1. Introduction

**Figure 1:**
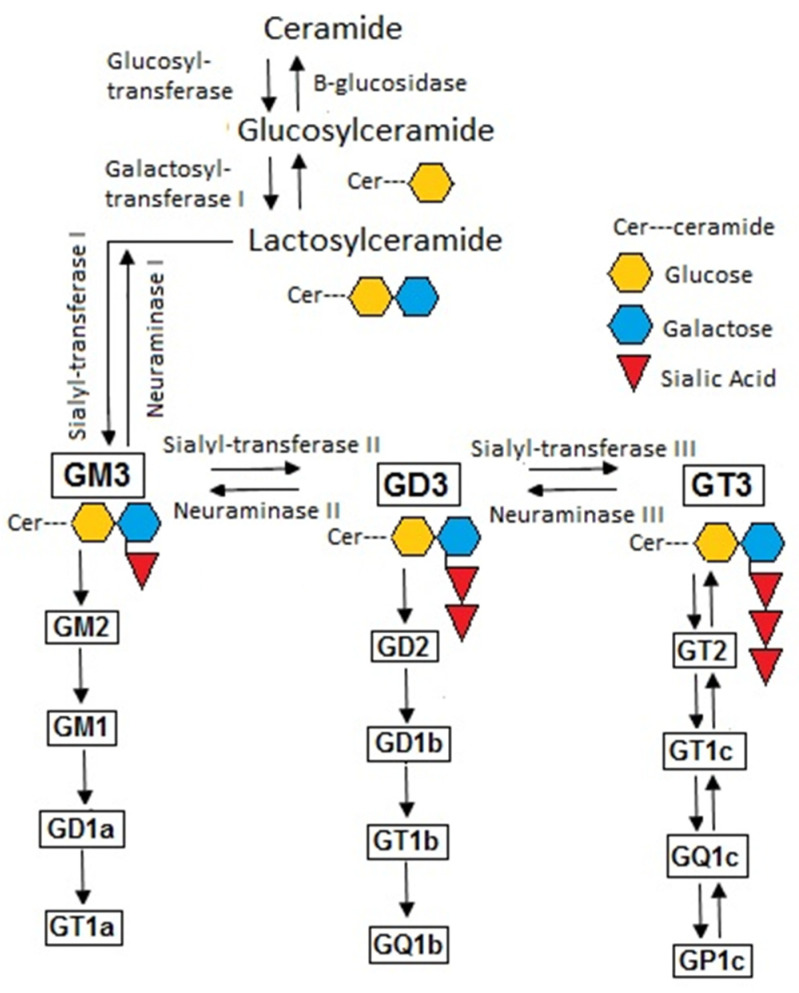
General Scheme for ganglioside biosynthesis. G indicates ganglioside; M indicates monosialo; D indicates disialo; T indicates trisialo; numbers indicate carbohydrate synthesis. The reactions of sialyl-transferases transfer sialic acid units. The reactions of neuramidases remove sialic acid units.

Gangliosides (GD1, GD3, GM3) are a subgroup of glycosphingolipids distinguished by the presence of one or more sialic acid units [1]. Ganglioside is biosynthesized from ceramide in the endoplasmic reticulum [2]. Addition of sialic acid units by sialyltransferases occurs in the Golgi apparatus and ganglioside is subsequently transported to the plasma membrane [2]. Factors determining the fatty acid components in the ganglioside, as well as the significance of different fatty acid constituents in the ganglioside, are largely unknown. GM3 synthase (STI, ST3Gal V, CMP-NeuAc:lactosylceramide-α2,3-sialyl transferase I) transfers sialic acid to lactosylceramide to form GM3 (Figure 1) [3]. GM3 is the simplest and most abundant ganglioside species present in intestinal mucosa, plasma, adipose tissue, liver skeletal muscle and brain and is the precursor of other gangliosides [3,4]. Gangliosides are located in the plasma membrane in microdomain structures such as lipid rafts or caveolae which are implicated in cell–cell recognition, adhesion, uptake and signal transduction [5]. In mice, GM3 is important in absorption of cholesterol [6].

Individuals with T2DM often have dyslipidemia, characterized by altered lipid profiles and overproduction of chylomicrons. The clearance rate of chylomicrons is slower than in healthy participants, potentially due to lower abundance of apolipoprotein E [7]. In T2DM, low grade inflammation may also contribute to dyslipidemia as Tumor Necrosis Factor-α (TNF-α) is associated with an increase of intestinal lipoprotein secretion that may elevate chylomicron production in an insulin resistant state [8,9]. In Caco-2 cells, TNF-α elevates the production of fatty acid binding proteins (FABPs) involved in chylomicron secretion [10]. The effect of T2DM on intestinal output of gangliosides is unknown.

Gangliosides can be acquired exogenously from diet or synthesized in the body [11]. Dietary gangliosides are absorbed by enterocytes causing increase of ganglioside content in intestinal mucosa, plasma, kidney, lung, liver and brain [4,12]. Human intestinal tissue and cultured cells take up gangliosides in vitro [13]. Chylomicrons are secreted by enterocytes during absorption of dietary fat and delivery of lipids to tissues [14]. Chylomicrons may potentially act as a vehicle for delivery of intestinal derived gangliosides to other tissues as GM3 has been found in chylomicrons and other lipoproteins in Watanabe hereditable hyperlipidemic rabbits [15].

GM3 content of serum and plasma gangliosides in people diagnosed with metabolic disorders was characterized [16]. Depletion of GM3 synthase in mice attenuated insulin resistance and inflammatory markers [17]. In human serum, GM3 (d18:1/h24:1) is highly correlated with the development of T2DM [18], however, no studies have examined the composition of plasma and chylomicron gangliosides in T2DM during the postprandial period. The objective of the present study is to determine the content and composition of GD1, GD3 and GM3 gangliosides in plasma and chylomicrons at fasting and 4 h postprandial when plasma chylomicrons are elevated in control and T2DM subjects. This comparison was made when each subject consumed a low or high fat diet in a crossover design.

## 2. Materials and Methods

### 2.1. Participant Characteristics

This study was approved by the Human Research Ethics Biomedical Panel at the University of Alberta. Samples for this study were collected as part of another study initiated before registration of clinical trials was standard [19].

Participants were recruited from the outpatient Metabolic Clinic at the University of Alberta Hospital and a list of respondents from a past study. All participants provided informed consent. Ten non-diabetic individuals (controls) and eleven subjects with T2DM were recruited. Exclusion criteria were triglyceride (TG) levels >4.0 mmol/L and subjects taking lipid lowering drugs. Subjects taking medications (metformin, levothyroxine and hypertensive drugs) continued their normal medications. Baseline screening results are presented (Table 1). Tests, diagnosis of type 2 diabetes and medications were reviewed by an endocrinologist.

### 2.2. Study Design

Seven subjects in each group completed all aspects of this study protocol. This study followed a 3 consecutive day crossover design separated by a one-month washout period. Intake, blinding and randomization of diet treatment order were controlled for this study. On days 1 and 2, prior to the test day, participants consumed either a high or low fat diet. The alternate diet was initiated one month later to start the second crossover study period. Participants recorded all food, drink and medications for seven days before initiating the high or low fat diet period. Participants picked up all meals and returned unwashed food containers as proof of compliance. After a fasting blood draw on day 3, the test day, breakfast was consumed. Twelve hour fasting blood samples were collected and postprandial blood samples were collected 4 h after consuming the test breakfast. Blood was collected into tubes containing lithium heparin for analysis of plasma lipids, glucose and insulin. Samples were analyzed by the University of Alberta Hospital Laboratory using automated standardized enzymatic procedures.

### 2.3. Diet Characteristics

Diets were designed to be isocaloric providing approximately 2100 kcal per day and differed primarily in total and monounsaturated fat content, in exchange for energy from carbohydrates. The Harris-Benedict equation and activity factor was used to calculate individual daily energy requirements, and the amount of diet was adjusted accordingly. The low fat diet contained 23%, 18% and 59% of energy from fat, protein and complex carbohydrate, respectively with limited simple sugar. The low fat diet (23% fat) contained saturated, monounsaturated, polyunsaturated and other fatty acids (5%, 6%, 10% and 2%, respectively). Both diets contained limited simple sugars. The high fat diet contained 37%, 16% and 48% of energy from fat, protein and carbohydrate, respectively. The high fat diet contained saturated, monounsaturated, polyunsaturated and other fatty acids (6%, 20%, 8% and 3%, respectively).

Both diets contained similar food items: breakfast was comprised of orange juice, blueberry oat bran muffins, scrambled egg and bread; lunch contained whole wheat pasta, tomato-based pasta sauce (vegetables, beef, mozzarella), peas, apple; and for dinner a turkey sandwich, raspberry newtons and melon pieces were provided. Safflower oil, flax oil and margarine with 50% less fat was added to the low-fat diet whereas canola oil and canola margarine was added to the foods in the high fat diet to achieve desired content and composition. The test meal consisted of the same breakfast meal consumed on the previous 2 days (low or high fat) and contained 1/3 of the individual’s daily caloric requirements. The amount of ganglioside in the test meal is estimated to be approximately 45–108 ug. The ganglioside content provided by the test meal is constant on an energy basis for each subject [20].

### 2.4. Sample Preparation and Chylomicron Collection

After collection, an aliquot of blood was centrifuged at 1000× *g* (Jouan CR 4.11 refrigerated centrifuge) for 10 min at 4 °C to separate plasma. Lipoprotein particles from 2 mL of plasma were separated using ultracentrifugation within 24 h of collection. Chylomicrons and chylomicron remnants were removed in a single centrifugation as described by Musliner et al**.**, 1991 [21]. and Layne et al**.**, 1996 [22]. The purity of the lipoprotein fractions separated was checked by electrophoresis to ensure that fractions were not cross-contaminated [22]. Samples were stored at −80 °C until analysis.

### 2.5. Liquid Chromatography/Triple Quad Mass Spectrometry Analysis

For ganglioside analysis, a modified Folch extraction was performed on 250 μL of plasma or lipoprotein fraction [23] using LC/MS grade solvents (Fisher Scientific Company, Ottawa, ON, Canada). A modified Folch extraction was performed with direct injection into the LC/triple quad MS as described earlier [24]. Prior to LC/triple quad MS analysis, the combined extract was reconstituted in water:methanol (1:1). For chylomicron analysis, the extract was divided into two samples. The first sample was prepared with 120 μL of 50/50 water and methanol, and the second sample with 50 μL hexane, 70 μL of 70% methanol, 15% water and 15% isopropyl alcohol prior to injection into the mass spectrometer. Two solvent systems were used as the solvent systems differed in polarity and, therefore, prevented clogging of the LC/triple quad MS due to extraneous triglyceride from the chylomicron fraction.

Gangliosides, GM3, GD3 and GD1 were analysed using LC/triple quad MS described by Rivas-Serna et al. (2015) [24]. Ganglioside masses were calculated assuming a sphingosine of d18:1 and d18:0. The prefix d indicates a dihydroxy base. For quantitative analysis, GM3 and GD3 ganglioside standards were purified and separated by TLC silica gel G plates (20 × 10 cm, 1000 μm, Analtech Inc., Newark, DE, USA). The solvent system used for TLC separation of individual gangliosides was chloroform/methanol/28%(*w*/*v*)ammonia/water (60:35:7:3, by volume) using ACS grade solvents (Fisher Scientific Company, Ottawa, ON, Canada) [24]. Gangliosides were visualized using 0.1% ANSA (Sigma-Aldrich, St. Louis, MO, USA). Standard gangliosides were prepared and quantified using a resorcinol–HCl assay (Sigma-Aldrich, St. Louis, MO, USA) [25]. Gangliosides were quantified relative to an external standard. The relative abundance of individual gangliosides was calculated from the total ganglioside species present (GM3, GD3, GD1). GM3, GD3 and GD1 in chylomicrons were obtained using the same method used to detect plasma ganglioside. Retention time and mass to charge ratio was used to determine if the peak was due to a ganglioside.

### 2.6. Statistical Methods

Data is expressed as mean ± SD. Statistical analyses were performed using SAS (Version 9.3, SAS Institute Inc., Cary, NC, USA). Significant difference between collection timepoints, diet fat level and two groups (T2DM and control) and interactions among different variables was determined by two-factor repeated measures of ANOVA with Duncan’s multiple range test as a post hoc test. Significant differences between high and low fat diets and individuals with T2DM and non-diabetic individuals were determined by Student T-test. Differences were considered statistically significant at *p* < 0.05.

## 3. Results

Participants were included if they consumed both diets and complied with study guidelines. A total of fourteen overweight/obese participants were included *n* = 7 per group. Potential participants were excluded due to illness, consuming lipid lowering drugs or completing only one dietary period. Groups were well-matched for sex, age and BMI (Table 1).

### 3.1. Dietary Intake

Unconsumed food was weighed to calculate the energy amount to be deducted from the second diet for three participants who did not consume all food prescribed in the first diet period. In the second diet period, all food was consumed by participants. After study completion, a dietary blinding success questionnaire was administered. Four participants identified both diets correctly.

### 3.2. Effect of Dietary Fat on Ganglioside Concentration in Plasma and the Chylomicron Fraction

Fat level in the diet had no effect on total or individual ganglioside content in plasma or chylomicron fractions during the postprandial period (*p* > 0.1, Table 2). Therefore, values for high and low fat treatments were combined in subsequent comparisons.

### 3.3. Effect of Fasting and 4 h Postprandial State on Plasma Ganglioside Content in Control and Diabetic Subjects

**Figure 2:**
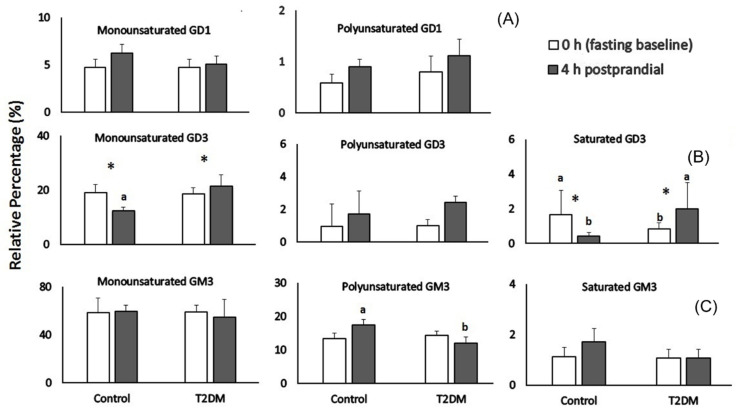
Gangliosides present in plasma at fasted and 4 h postprandial. (**A**) Percentage of mono-, poly-unsaturated GD1 ganglioside species in plasma found at fasting (baseline) and 4 h postprandial in control and diabetic subjects. (**B**) Percentage of mono-, poly-unsaturated and saturated GD3 species in plasma found at fasting baseline and 4 h postprandial timepoint in control and diabetic subjects. (**C**) Percentage of mono-, poly-unsaturated and saturated GM3 ganglioside species in plasma observed at fasting baseline and the postprandial in control and diabetic subjects. Low and high fat diet treatments were combined in subsequent analysis since no significant difference was detected in these groups. Data represent mean ± SD. Letters (^a,b^) indicate significant difference at *p* < 0.05 when individual ganglioside species are compared between control and T2DM subjects and symbol (*) indicate significant difference at *p* < 0.05 when individual ganglioside species are compared at 0 and 4 h time periods in the same group. Monounsaturated GD3 species (*p* < 0.01) and polyunsaturated GM3 (*p* = 0.01).

For all subjects, GM3 was the most abundant ganglioside found in plasma samples followed by GD3 and GD1 (Table 3). Total ganglioside content, total GM3, GD1, GD3 content in plasma did not change during the postprandial period in either control or diabetic subjects (*p* > 0.2; Table 3). In all subjects, monounsaturated ganglioside species were the most abundant species in plasma and contributed to 77–83% of total gangliosides. The relative percentage of polyunsaturated species represented between 15–20%. Saturated ganglioside species were the least abundant (2–3%) (Figure 2). The composition of the gangliosides changes over the postprandial period between T2DM and control subjects (Figure 2).

The relative percentage of plasma monounsaturated and polyunsaturated GD1 did not change over the postprandial period between T2DM and control subjects (*p* > 0.05, Figure 2A). The relative percentage of plasma monounsaturated GD3 species decreased by 40% at 4 h postprandial compared to the fasting state in control subjects (*p* < 0.01, Figure 2B). In subjects with T2DM, monounsaturated GD3 species were 75% higher than control subjects after meal consumption (*p* < 0.01, Figure 2B). Plasma content of saturated GD3 species decreased 4 fold from 0 h by 4 h in control subjects (*p* < 0.05, Figure 2B) and increased 140% after meal consumption when compared to fasting state in diabetic subjects (*p* < 0.05, Figure 2B). Plasma polyunsaturated GD3 species were not affected by postprandial period nor diabetic state (*p* > 0.2, Figure 2B).

The relative percentage of plasma polyunsaturated GM3 species was 30% higher in control subjects at 4 h compared to diabetic subjects (*p* = 0.01, Figure 2C). Monounsaturated and saturated GM3 species did not change during the postprandial period (Figure 2C).

### 3.4. Effect of Type 2 Diabetes and Postprandial Period on Total GD1 in Chylomicrons

**Figure 3:**
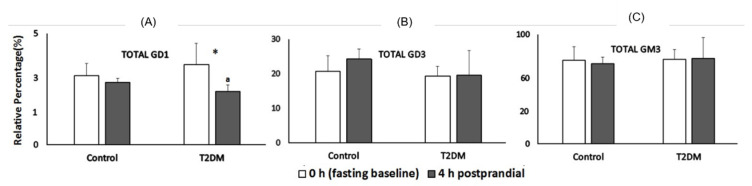
Gangliosides present in chylomicrons at fasted and 4 h postprandial. (**A**) Relative percent of total GD1 in chylomicron fraction at fasting and 4 h postprandial in control and T2DM groups (*p* = 0.01). (**B**) Relative percent of total GD3 in chylomicrons at fasting and 4 h postprandial in control and T2DM groups. (**C**) Relative percent of total GM3 in chylomicrons at fasting and 4 h postprandial in control and T2DM groups. Low and high fat diet treatments were combined in subsequent analysis since no significant difference was detected in these groups. Total GD1 = monounsaturated GD1 species. Total GD3 = saturated and monounsaturated GD3 species. Total GM3 = saturated, monounsaturated and polyunsaturated GM3 species. Data represent mean ± SD. Letters (^a,b^) indicate significant difference at *p* < 0.05 when individual ganglioside species are compared between control and T2DM subjects and symbol (*) indicate significant difference at *p* < 0.05 when individual ganglioside species are compared at 0 and 4 h time periods in the same group.

The most abundant ganglioside in the chylomicron fraction was GM3, followed by GD3 and GD1 (Figure 3). Total GD1 (comprising monounsaturated GD1 species) did not change during the postprandial period in control subjects whereas total GD1 decreased by 50% at 4 h postprandial in subjects with T2DM (*p* = 0.01, Figure 3A). Total GD3 (Figure 3B) and GM3 (Figure 3C) did not change during the postprandial period in control or diabetic subjects.

### 3.5. Effect of Type 2 Diabetes and Postprandial Period on Mono-Unsaturated and Saturated GD1, GD3 and GM3 Species in Chylomicrons

GD3 d34:1 content was 50% more abundant in diabetic subjects compared to control subjects at baseline (*p* = 0.008, Table 4), but was similar at 4 h postprandial. The content of GD1 d36:1 was 35% lower after meal consumption in T2DM subjects compared to controls (*p* = 0.002, Table 4). In control subjects, the relative content of GM3 d36:0 decreased approximately 66% postprandially (*p* = 0.01, Table 4), but was unchanged in diabetic subjects.

## 4. Discussion

The present study identifies specific changes occurring in ganglioside content and hence, metabolism, in plasma and chylomicrons in people with T2DM during postprandial periods. Postprandial changes were observed in monounsaturated and saturated GD3 ganglioside in control and T2DM participants. Recently, it was found that in healthy women, some individual GM3 species changed in concentration but total GM3 ganglioside did not change over the 8 h postprandial course [26]. However, diabetes seems to influence the ceramide composition of GM3 and other ganglioside species secreted from the intestinal mucosa. In the present study, plasma ganglioside content was affected by the postprandial period and by type 2 diabetes. Four hours after meal consumption, subjects with T2DM exhibited less abundant polyunsaturated GM3 species and more abundant monounsaturated and saturated GD3 ganglioside in plasma compared to control subjects. This alteration in plasma ganglioside content and composition in T2DM may reflect the fate of gangliosides during the postprandial period through catabolic and anabolic processes altering the balance between pro-inflammatory and anti-inflammatory gangliosides.

Plasma gangliosides may show an anabolic conversion between GM3 to GD3 with no significant alteration occurring in GD1; the effect of conversion between GM3 to GD3 is apparently affected in T2DM resulting in altered polyunsaturated GM3 and monounsaturated and saturated GD3 species. The association between fasted serum ganglioside concentrations in healthy controls, compared to subjects with hyperglycemia, hyperlipidemia, or both was assessed [16]. Serum levels of GM3 were 60% higher in individuals with hyperglycemia concurrent with hyperlipidemia and 40% higher in subjects with hyperglycemia only compared to healthy subjects. Individuals with T2DM and obesity exhibited elevated serum GM3 levels compared to non-obese diabetic patients and suggest that glucose and lipid disorders as well as visceral adiposity contribute to higher levels of serum GM3 levels [16]. Visceral fat was not directly evaluated in the current study; participants had central obesity as assessed by waist circumference but were matched on this basis.

The enterocyte is an active participant in ganglioside metabolism. Feeding rats a GD3 enriched diet increased GD3 content in intestinal mucosa lipid rafts and plasma [4]. Gangliosides are absorbed in the enterocyte and cross the brush border to raise plasma ganglioside levels [4,11]. Plasma ganglioside is not only a result of secretion of ganglioside in chylomicrons, but may also reflect synthesis of ganglioside in other tissues such as the liver. In fasted, healthy subjects, gangliosides are predominantly transported by lipoproteins (LDL, HDL, VLDL) and biosynthesized in the liver [27,28].

Gangliosides contain a ceramide portion influencing the structure of membrane rafts and caveolae [29]. GM3 apparently regulates ganglioside content in all cells and is involved in signal transduction processes [3]. Dietary GD3 and GM3 gangliosides have been related to improved immune functions and permeability in the gut [30]. Low intensity chronic inflammatory diseases such as diabetes may alter metabolism of sphingolipids including ceramides [31]. TNF-α activates GM3 synthase gene in 3T3-L1 adipocytes and the addition of exogenous GM3 inhibits the phosphorylation of insulin receptor [32]. The depletion of GM3 synthase in skeletal muscle of mice consuming a high fat diet improves insulin resistance and glucose uptake [33]. Increase of GM1 and GM2 species inhibits phosphorylation of insulin receptor in diabetic mice, whereas GM3 and GD1a does not affect insulin signaling [34]. In mice it has been reported that alteration of ganglioside synthesis may affect glucose uptake by islet cells [35]. It is noteworthy that while the TG level increased in the T2DM group, the level of total ganglioside remained similar to the ganglioside level in the control group. Further research is needed to evaluate the metabolic role of ganglioside species in T2DM. Ceramides have been associated with glucose homeostasis, insulin signaling processes and the diabetic phenotype [36]. In animal models, C16 carbon chain ceramides have been reported to be involved in insulin signaling and may contribute to obesity and insulin resistance [37]. In mice, the metabolism of neutral ceramidase (nCDase, N-acylsphingosine amidohydrolase 2, ASAH2) is involved in metabolism of sphingolipids and exogenous ASAH2 treatment improves insulin sensitivity [38,39]. The limitations of the study are that the method used does not distinguish between GD1a and GD1b ganglioside species. Further studies should include modification of the methods to determine the difference between GD1a and GD1b. Further studies should also consider a lean normal control group for comparison.

## 5. Conclusions

These observations combined with the present results indicate that gangliosides are important components involved in the metabolic mechanisms of type 2 diabetes and that the intestinal mucosa has a significant role in this aspect of interorgan metabolism. Moreover, the ceramide composition of GD1, GD3 and GM3 in plasma and chylomicrons may have a unique contribution to the postprandial response in the type 2 diabetic state.

## Figures and Tables

**Table 1:** Baseline characteristics of participants.

Group	Control	T2DM
Gender (M/F)	3M/4F	3M/4F
Age (year)	51.4 ± 9.2	50.0 ± 8.8
BMI (kg/m^2^)	33.5 ± 8.3	33.2 ± 7.5
Weight (kg)	93.4 ± 24.1	91.9 ± 15.4
Waist (cm)	105.6 ± 15.8	105.6 ± 13.5
Glucose (mmol/L)	5.0 ± 0.4	6.2 ± 1.1 *
HBAlc (%)	5.3 ± 0.4	5.9 ± 0.5 *
Insulin (pmol/L)	69.0 ± 53.0	95.0 ± 55.0
HOMA-IR	2.2 ± 1.7	3.9 ± 2.4
FFAs (mmol/L)	0.9 ± 0.4	0.7 ± 0.3
Triglycerides (mmol/L)	1.3 ± 0.4	2.0 ± 0.8
TC (mmol/L)	5.4 ± 0.8	4.8 ± 0.6
HDL-C (mmol/L)	1.3 ± 0.2	1.2 ± 0.2
LDL-C (mmol/L)	3.5 ± 0.7	2.7 ± 0.6 *
C-reactive protein (mg/L)	0.6 ± 0.3	1.6 ± 1.2

Mean ± SD. * indicates significant difference (*p* < 0.05) between control and T2DM group (*n* = 7 per group). Abbreviations: T2DM, type 2 diabetes mellitus; M, male; F, female; BMI, body mass index; HBA1c, Hemoglobin A1c; HOMA; homeostatic model assessment; FFAs, free fatty acids; TC, total cholesterol; HDL, high density lipoproteins; LDL, low density lipoproteins.

**Table 2:** Total GM3, GD3, GD1 content in plasma and chylomicrons after consuming a low and high fat diet.

Ganglioside Content	High Diet Fat Relative Percentage (%)	Low Diet Fat Relative Percentage (%)	*p*-Value
Plasma GM3 Plasma GD3 Plasma GD1 Chylomicron GM3 Chylomicron GD3 Chylomicron GD1	70.7 ± 3.1 23.3 ± 3.4 6.0 ± 0.7 77.1 ± 3.0 19.8 ± 3.2 3.1 ± 0.5	76.0 ± 1.4 17.8 ± 1.3 6.2 ± 0.6 74.9 ± 3.9 22.3 ± 4.1 2.8 ± 0.4	0.1 0.3 0.9 0.7 0.6 0.6

Data are expressed as mean ± SD. No significant difference was found between low and fat level in the diet (*p* > 0.05).

**Table 3:** Concentration (ng/mL) of GM3, GD3, GD1 and total ganglioside at fasting and in postprandial plasma samples.

Ganglioside Species	Time (h)	Control (ng/mL)	T2DM (ng/mL)
GM3	0	681.7 ± 89.0	641.7 ± 67.0
	4	648.9 ± 59.0	667.5 ± 76.5
GD3	0	26.8 ± 7.9	20.9 ± 3.1
	4	23.7 ± 3.1	27.6 ± 5.5
GD1	0	11.2 ± 2.8	9.7 ± 3.2
	4	14.8 ± 2.6	12.8 ± 4.3
Total	0	719.7 ± 97.6	672.3 ± 72.1
	4	679.4 ± 63.2	707.9 ± 58.0

Data are expressed as mean ± SD. No significant difference was found between control and T2DM groups at different time periods (*p* > 0.05). Abbreviations T2DM, type 2 diabetes mellitus.

**Table 4:** Relative percent of individual ganglioside species in chylomicrons at 0 and 4 h postprandial in control and T2DM patients.

Individual Ganglioside Species	Time	Control Relative Percentage (%)	T2DM Relative Percentage (%)
GD3 d34:1	0 h	6.0 ± 0.2 ^a^	9.1 ± 0.3 ^b^
	4 h	7.8 ± 0.3	6.4 ± 0.3
GD1 d36:1	0 h	2.2 ± 0.3	2.7 ± 0.1
	4 h	2.9 ± 0.1	1.1 ± 0.2 *^,a^
GM3 d36:0	0 h	0.3 ± 0.02	0.2 ± 0.03
	4 h	0.1 ± 0.01 *^,a^	0.4 ± 0.04 ^b^

Data are expressed as mean ± SD. Letters (^a,b^) indicate significant difference at *p* < 0.01 when individual ganglioside species are compared between control and T2DM subjects and symbol (*) indicate significant difference at *p* < 0.01 when individual ganglioside species are compared at 0 and 4 h time periods in the same group.

## Data Availability

The data presented in this study are available on request from the corresponding author.

## Abbreviations

T2DM	Type 2 diabetes mellitus
LC/MS	Liquid chromatography/Mass spectrometry
TLC	Thin Layer Chromatography
